# Synergistic Effect of Anemia and Red Blood Cells Transfusion on Inflammation and Lung Injury

**DOI:** 10.1155/2012/924042

**Published:** 2012-08-07

**Authors:** Anping Dong, Manjula Sunkara, Manikandan Panchatcharam, Abdel Salous, Samy Selim, Andrew J. Morris, Susan S. Smyth

**Affiliations:** ^1^Division of Cardiovascular Medicine, The Gill Heart Institute, 741 S. Limestone Street, 252 BBSRB, Lexington, KY 40536-0509, USA; ^2^Medical Service, Lexington VA Medical Center, 1101 Veterans Drive, Lexington, KY 40502, USA

## Abstract

Anemia and resultant red blood cell transfusion may be associated with adverse long-term clinical outcomes. To investigate the mechanism(s) responsible, we profiled inflammatory biomarkers and circulating levels of the bioactive lysophospholipid mediator sphingosine-1-phosphate (S1P) in control and anemic mice with or without LPS-induced systemic inflammation. Acute anemia or lipopolysaccharide (LPS) challenge alone triggered an increase of circulating levels of the inflammatory markers IL-6 and keratinocyte-derived chemokine (CXCL1/KC). Moreover, administration of LPS to anemic mice reduced circulating S1P levels and augmented lung injury and pulmonary vascular permeability. Transfusion of aged, but not fresh, red blood cells (RBCs) worsened pulmonary vascular leak. S1P levels decline markedly during storage of mouse RBCs. Loading stored murine RBCs with S1P prior to transfusion partially attenuated anemia-associated acute pulmonary vascular leak. Taken together, our results indicate that anemia and systemic inflammation can alter the S1P buffering capacity of RBCs, suggesting possible strategies for alleviating transfusion-related lung injury in clinical practice.

## 1. Introduction

Blood or packed erythrocytes are commonly administered to individuals with anemia, especially in settings of trauma and critical illness. While acutely lifesaving, transfusions may trigger events that contribute to, rather than prevent, long-term morbidity and mortality [1–4]. In patients with acute respiratory distress and multiorgan failure, red blood cell (RBC) transfusion can promote acute lung injury and may predict mortality. The adverse effects of transfusion are hypothesized to require two “hits”—one relating to inflammatory or additional predisposing condition(s) in the recipient and the second involving alterations in the transfused blood. For the later, the age of the transfused blood may be important, as alterations during storage may occur. 

The factor(s) responsible for tissue injury in the setting of transfusion remain to be identified. Disruption in nitric oxide availability, fragility and impaired deformability of the erythrocyte plasma membrane, and generation of microparticles and bioactive lipids have been proposed as underlying causes of the storage lesion. Any one—or a combination of these phenomena—could produce systemic inflammation, disrupt the integrity of the endothelial barrier, and promote tissue edema and leukocyte infiltration in susceptible recipients [5]. A recent study indicates that transfusion of fresh RBC counteracts the adverse effects of aged RBC on systemic inflammation [6], implying that older RBCs have lost a protective factor during storage, although the identification of the factor remains unknown.

Erythrocytes have been identified as an important buffer for the bioactive lipid mediator, sphingosine-1-phosphate (S1P) [7], an *in vivo* regulator of endothelial permeability and immune cell function. In mice, depletion of plasma S1P by genetic inactivation of S1P synthesizing enzymes (sphingosine kinases 1 and 2) elicits profound pulmonary vascular leak, which can be reversed by restoring circulating S1P via RBC transfusion. In humans, hematocrit (Hct) predicts plasma S1P levels. However, we previously reported that in anemic individuals, plasma S1P levels are not uniformly restored by RBC transfusion. Rather, the age of the RBC unit at the time of transfusion tended to negatively correlate with the ability of RBC transfusion to replenish plasma S1P [8]. During storage, the S1P content of human RBC markedly declines, likely due to enzymatic degradation [8]. Because erythrocytes serve as a buffer for circulating S1P, aged RBC with low S1P content may be incapable of restoring plasma S1P levels and may actually remove S1P from plasma, which in turn could contribute to increased endothelial permeability, capillary leak, and infiltration of inflammatory cells. However, while S1P appears to inversely correlate with age of RBC unit, whether it serves as a protective factor in fresh RBCs [6] is not known.

In this study, we established the effects of transfusion of both fresh and aged RBC on systemic inflammation and lung permeability in a model in which anemia is combined with a low-level inflammatory challenge in mice. We investigated whether blood and plasma S1P levels associated with pathophysiologic changes and evaluated the ability of S1P-loaded RBC to attenuate tissue injury. Our findings could be clinically important and may represent a future, novel therapeutic strategy for vascular barrier dysfunction. 

## 2. Materials and Methods 

### 2.1. Animals

Male mice (C57BL/6J) aged eight to 10 weeks were purchased from The Jackson Laboratories (Bar Harbor, ME). Mice were acclimated for at least one week prior to our procedures and used in experimental protocols when their weights were *≈*25 g. Mice were fed standard rodent chow (Purina 5058) ad libitum and were maintained on 14-hour day/10-hour night light cycles. All procedures were approved by the Institutional Animal Care and Use Committee. 

### 2.2. Acute Anemia Model

C57BL/6J mice were made anemic by phlebotomy (0.02 mL/gm) following the protocol of Sadahira et al. [9] and then were immediately given an equal volume of sterile normal saline via intraperitoneal injection to maintain plasma volume. To distinguish between anemia and vascular trauma from phlebotomy, selected mice were phlebotomized but subjected to minor blood loss (0.002 mL/gm) where indicated. Twentyfour to 48 hours after the initial blood loss, an additional volume of blood was collected into EDTA-coated tubes for complete blood count analysis (CBCs) and performed with an ABC vet hematology analyzer, blood and plasma S1P, and plasma inflammatory biomarkers. Plasma cytokines were quantified using a mouse multiplex assay kit (MPXMCYTO-70 K; Millipore, Billerica, MA) according to the manufacturer's 96-well plate assay protocol. Bead fluorescence was detected using a BioRad Bio-Plex 200 suspension array reader (BioRad Laboratories, Hercules, CA) and analyzed using Bio-Plex Manager 4.0 software (BioRad Laboratories). Sphingosine-1P was measured by LC/MS/MS from samples (50 *μ*L) of plasma, whole blood, and packed RBCs after extraction of lipids in MeOH/CHCL_3_/HCl either using C17-LPA as a recovery standard with an offline calibration, or by stable isotope dilution using d7 S1P as the internal standard.

### 2.3. RBC Isolation and Storage

Packed RBCs were prepared by modification of protocols established by the American Association of Blood Banks. Blood was collected from isoflurane-anesthetized mice into a sterile tube containing sterile citrate phosphate dextrose solution (CPD: Sigma-Aldrich, St. Louis, MO) at a ratio of 6 parts blood:1 part CPD. The blood was centrifuged at 5000 g for eight minutes at room temperature, and the platelet-rich plasma and buffy coat were removed. The RBC pellet was resuspended in sterile phosphate buffer solution (PBS; Baxter Healthcare Corporation, Deerfield, IL). RBCs were isolated on an autoclaved microcellulose column (Sigma-Aldrich) following the protocol of Beutler et al. [10] to remove >99% of leukocytes. The eluent was centrifuged to remove residual plasma and platelets. The erythrocyte pellet was stored in sterile AS-5 (Terumo, Somerset, NJ) at 4°C at a ratio of 5 parts RBC:1 part AS-5 until transfusion. Immediately prior to transfusion, RBCs were resuspended in sterile PBS at 50% Hct. Fresh RBCs were administered to mice on the day of preparation; aged RBC were stored for >14 days at 4°C before transfusion. 

To load RBCs with S1P, cells were washed by centrifugation in Tyrode's buffer containing 0.3% benzenesulfonic acid to remove AS-5. RBCs in Tyrodes containing 0.3% BSA were incubated at 37°C for 60 minutes with 0.5 mM C17-S1P or S1P, washed twice by centrifugation, resuspended in sterile PBS at a 50% Hct, and immediately administered to mice. 

### 2.4. RBC Transfusion

Twentyfour hours after the initial blood collection, mice were anesthetized with isoflurane and randomly assigned to experimental groups. One group received 0.3 mL fresh RBC (50% Hct in PBS), the second received 0.3 mL of aged RBC (50% Hct in PBS), and the control group received 0.3 mL sterile PBS. Intravenous injections were performed via jugular vein with a 29-gauge needle. RBCs were warmed to room temperature before transfusion. 

### 2.5. LPS Challenge

Mice were injected intraperitoneally with 2 mg/kg lipopolysaccharide (LPS, *Salmonella enteritidis*), in sterile 0.9% saline or an equal volume of 0.9% saline as control at 24 hours after blood loss. 

### 2.6. Pulmonary Vascular Permeability

Mice were anesthetized with inhaled isoflurane. One hundred *μ*l of 1% Evans blue dye (EBD) in PBS was injected into the right jugular vein. Fifteen minutes later, mice were euthanized and perfused with PBS through the right ventricle at a constant rate to achieve 25 mmHg pressure. The lungs were dissected, weighed, and immersed in a 1 mL 4% formamide overnight. The OD at 620 nm of the solution was recorded and normalized to lung weight. In some experiments, EBD in the intact lung was quantified by fluorescence measurements using an Odyssey infrared laser scanner (LI-COR, Lincoln, NE) and normalized to lung weight. 

### 2.7. Statistics

All results were expressed as mean ± SD. The results were analyzed by Student *t*-test or analysis of variance (ANOVA). Statistical analysis was performed using Sigma-STAT software version 3.5 (Systat Software Inc., Chicago, IL). A probability value of less than 0.05 was considered significant. 

## 3. Results

### 3.1. Effects of Blood Loss on Inflammation and Bioactive Markers in Circulation

**Figure 1:**
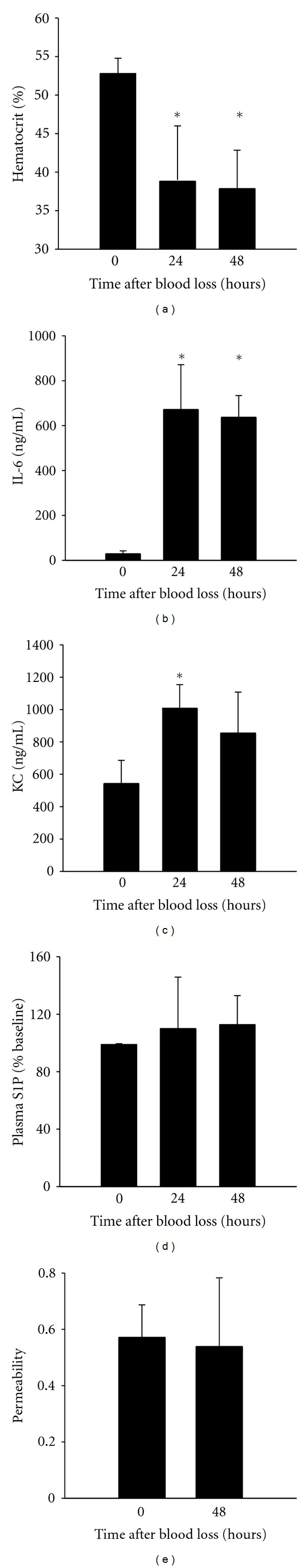
Effect of blood loss on systemic inflammation response and lung endothelial permeability. (a) Hct, (b) plasma IL-6, (c) plasma KC, and (d) plasma S1P were measured at baseline (time = 0; *n* = 6) or at 24 (*n* = 3) or 48 hours (*n* = 3) after major blood loss (0.02 mL/gm). (e) Lung permeability was measured in mice that had not undergone blood loss (time = 0; *n* = 3) or at 48 hours after major blood loss (*n* = 3) by quantifying accumulation of IV injected EBD into the lung. Results were analyzed by one-way ANOVA (Bonferroni correction) *P < 0.05 versus time = 0.

We profiled the impact of vascular trauma and blood loss on circulating inflammatory biomarkers in mice. At 24 and 48 hours after major blood loss (0.02 mL/gm body weight), corresponding to an *≈*20% absolute decline in Hct (Figure 1(a)), circulating levels of the inflammatory markers IL-6 (Figure 1(b)) and CXCL1/KC (Figure 1(c)) increased significantly (*P* < 0.05). Plasma levels of IFN*γ*; MCP-1, RANTES, TNF*α*, IL-10, or MIP-2 were unchanged after blood loss (see Supplementary Figure 1 in Supplementary material available online at doi:10.1155/2012/924042). The effects of blood loss on plasma IL-6 were proportional to the volume of blood collected (Supplementary Figure 2), with an 8.6-fold lower plasma IL-6 observed after collection of half the blood volume (0.01 mL/gm body weight). Moreover, plasma IL-6 was undetectable 48 hours after collection of a small volume of blood (0.002 mL/gm or 0.05 mL), indicating that vascular trauma from phlebotomy *per se* did not elicit a detectable inflammatory response. Erythrocytes serve as a reservoir for cellular and extracellular S1P, an endothelial barrier stabilizing agent [7]. In humans, anemia is accompanied by a decrease in plasma S1P. Here, we found that plasma S1P levels did not decline in mice at 24 to 48 hours after blood loss (Figure 1(d)). Instead, following phlebotomy, total S1P per unit RBC volume increased, resulting in higher whole blood S1P levels (Supplementary Figure 3). Major blood loss alone did not alter lung endothelial permeability (Figure 1(e)).

### 3.2. Effects of Blood Transfusion following Blood Loss on Circulating Inflammatory Markers and S1P

**Figure 2:**
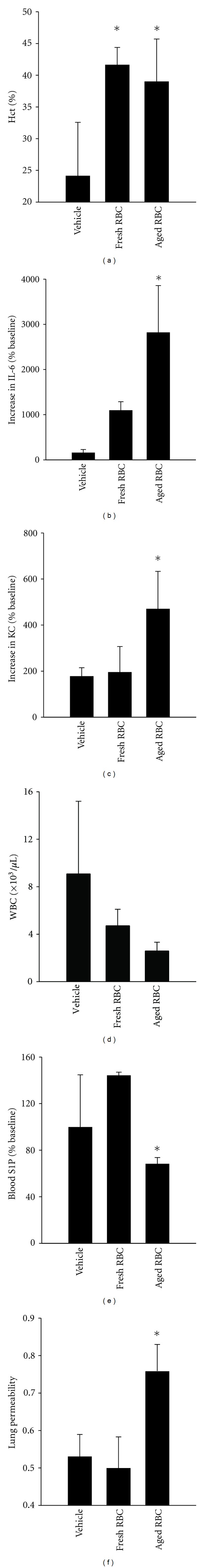
Effect of transfusion of fresh and aged RBC on systemic inflammation response and lung endothelial permeability. Twenty-four hours after major blood loss (0.02 mL/gm), mice were administered 0.3 mL of PBS (“vehicle”), freshly isolated RBC at 50% Hct (“fresh RBC”), or RBC stored for >14 days (“aged RBC”) at 50% Hct. Fortyeight hours after initial blood loss, (a) Hct, (b) plasma Il-6, (c) plasma KC, (d) white blood cell count (WBC), (e) blood S1P, and (f) lung endothelial permeability were measured. IL-6, KC, and S1P are reported as % of baseline (time 0). Results were analyzed by one-way ANOVA (Bonferroni correction),*P < 0.05 versus vehicle.

We next determined whether restoration of Hct by RBC transfusion 24 hours after blood loss had an impact on circulating inflammatory biomarkers or endothelial permeability. Administration of fresh (transfused on the day of isolation) or aged (stored for >14 days before transfusion) RBC improved Hct (Figure 2(a)). Transfusion of aged but not fresh RBC, increased plasma Il-6 and CXCL1/KC levels (Figures 2(b) and 2(c)), reduced white blood cell (WBC) count (Figure 2(d)), and modestly promoted lung endothelial permeability (Figure 2(f)). Following administration of fresh RBC, whole blood levels of S1P increased slightly, whereas they declined after administration of aged RBC (Figure 2(e)). To understand the reason for the differential effect of fresh and aged RBC on plasma S1P levels, we measured S1P content in RBC during storage and observed a steady decline over time (Supplementary Figure 4), such that the S1P content of erythrocytes stored for 30 days was <25% of that found in freshly isolated cells. Likewise, the dihdro-S1P (DHS1P) content of murine erythrocytes also decreased during storage (Supplementary Figure 4). These findings indicate that RBCs are 1 of at least 2 main sources for blood S1P in mice and that the S1P content of transfused RBCs affects blood S1P levels.

### 3.3. Effects of an Inflammatory Challenge following Blood Loss

**Figure 3:**
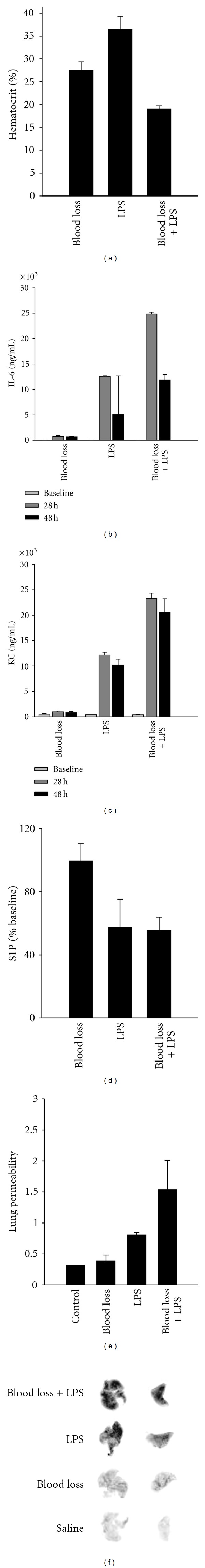
Effect of anemia on LPS-induced systemic inflammation and lung endothelial permeability. At time = 0 (baseline), mice were randomized in a 2 : 1 fashion to major blood loss (0.02 mL/gm) or minor blood collection (0.002 mL/gm). Twentyfour hours later, mice that had undergone major blood loss (“blood loss”) were given vehicle or low dose LPS (2 mg/kg, i.p.). Mice that had undergone minor blood loss were given LPS alone. (a) Hct was measured at 48 hours after initial blood loss (*n* = 3 mice/group). (b) Plasma Il-6 and (c) plasma KC were measured at 28 hours or 48 hours after initial blood loss (*n* = 3 mice/group). (d) Blood S1P was measured at 28 hours. (e) Quantification of EBD in the lungs, as a marker for endothelial permeability, was performed at 48 hours after initial blood loss (*n* = 3/group). (f) Representative images of EBD in the lungs after the indicated treatments. Results were analyzed by one-way ANOVA (Bonferroni correction); *P < 0.05 versus vehicle.

The adverse consequences of anemia and transfusion may manifest in settings of inflammation. Therefore, we sought to understand how acute inflammation in the setting of anemia impacts systemic responses. Administration of a low-dose LPS challenge (2 mg/kg) to animals 24 hours after blood loss was associated with lower Hct than observed in mice injected with saline alone (Figure 3(a)). LPS alone promoted the expected systemic inflammatory response, characterized by elevated plasma levels of IL-6 (Figure 3(b)) and CXCL1/KC (Figure 3(c)). This inflammatory response was magnified in mice with preceding blood loss (Figures 3(b) and 3(c)). LPS-induced thrombocytopenia occurred in both control and anemic mice and resulted in reduced platelet aggregation in whole blood (Table 1). Following LPS injection, both plasma and RBC S1P levels were slightly, but not significantly, lower than observed at baseline (Figure 3(d)). Lung leak at four hours after LPS injection was greater in mice that had previously sustained blood loss in comparison to control mice (Figures 3(e) and 3(f)). 

**Figure 4:**
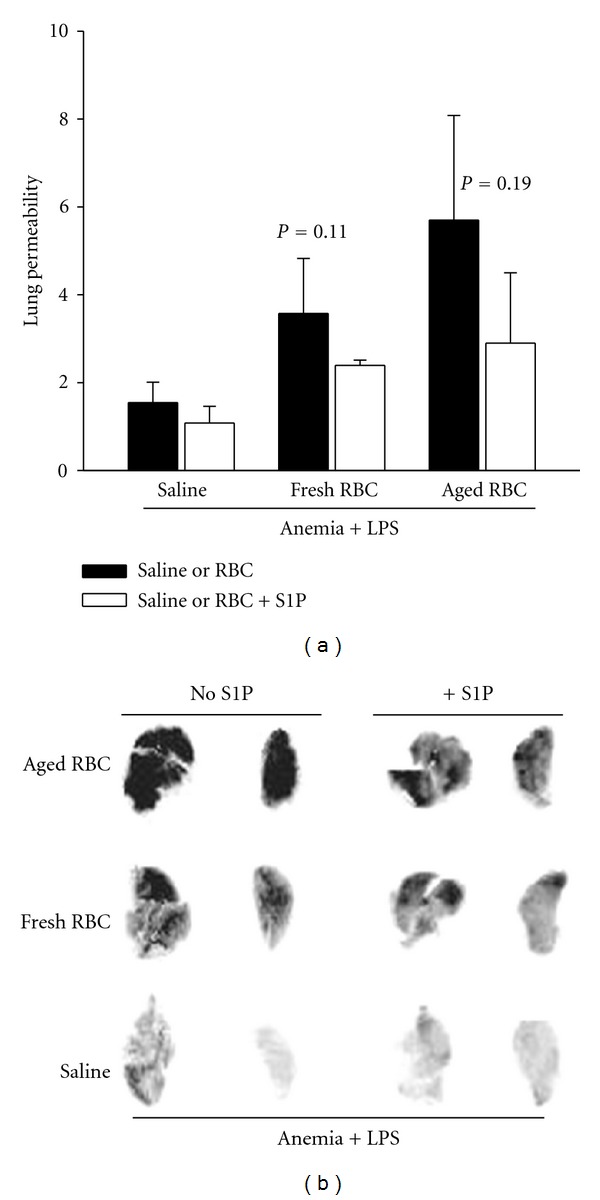
Effect of transfusion of S1P-loaded fresh and aged RBC on lung endothelial permeability.Twentyfour hours after major blood loss (0.02 mL/gm), mice were treated with LPS. Four hours after LPS treatment, fresh or aged RBCs with or without a preload with SIP (RBC + S1P) were transfused. (a) Evans blue dye (EBD) in the intact lung quantified and (b) representative images of the dye in the lungs demonstrated. Results were analyzed by one-way ANOVA (Bonferroni correction); *P < 0.05 versus vehicle.

Next, we determined whether RBC transfusion attenuated LPS-mediated endothelial barrier dysfunction in anemic mice. Rather than protecting against LPS-mediated lung permeability, fresh RBC transfusion given after LPS slightly heightened pulmonary vascular leak (Figures 4(a) and 4(b)). The adverse effect of transfusion on pulmonary vascular integrity was amplified when aged RBCs (stored for >14 days) were used (Figures 4(a) and 4(b)). 

In light of previous reports of a role for S1P in maintaining vascular permeability and the ability of RBC transfusion to restore plasma S1P in mice, we sought to determine if the adverse effects of transfusion could be overcome by loading the cells with S1P prior to transfusion. Human RBCs accumulate exogenously supplied S1P [11]. Similarly, S1P rapidly associated with isolated murine erythrocytes (Supplementary Figure 5(a)). Consistent with the observation that erythrocytes release S1P in the presence of albumin or lipoproteins, transfusion of S1P-loaded RBC increased plasma S1P levels in recipient mice (Supplementary Figure 5(b)). Loading either fresh or aged RBC with S1P prior to transfusion lowered lung permeability in the setting of LPS, although the results did not reach statistical significance (Figures 4(a) and 4(b)). 

## 4. Discussion 

 In this study, we investigated the consequences of RBC transfusion in the setting of anemia without or with a systemic inflammatory challenge in mice. Most previous preclinical models have not examined transfusion in the setting of anemia, nor have the consequences of transfusion in the background anemia and inflammation been described. In our model, blood transfusion was performed after blood loss alone, or in combination with an inflammatory challenge, to simulate the two “hits” thought to contribute to adverse effects observed in transfusion recipients. Acute, posthemorrhagic anemia was mimicked in mice by withdrawing *≈*20% of the calculated blood volume [9], and systemic inflammation was elicited by injecting LPS (2 mg/kg). We observed that the combination of anemia and LPS administration resulted in higher plasma levels of the inflammatory markers IL-6 and CXCL1/KC than did either challenge alone. Hematocrit improved similarly in anemic mice transfused with fresh or aged RBCs, but plasma IL-6 and CXCL1/KC were higher after transfusion of aged RBCs.

Because the association of worse clinical outcomes following transfusion of aged blood may relate in part to perturbation of lung endothelial barrier function, we also investigated lung permeability in our model. Neither anemia nor transfusion of fresh RBC in anemic mice altered lung permeability as assessed by extravasation of EBD, whereas transfusion of aged RBCs resulted in mild pulmonary vascular leak. LPS administration to anemic mice resulted in augmented pulmonary vascular permeability in comparison to nonanemic mice, suggesting that this is a reasonable model to provide mechanistic insight into events that may occur in anemic patients receiving transfusion that results in acute lung injury. 

Alterations in levels or actions of bioactive lipids have been proposed as contributing to the RBC storage lesion. Red blood cells are the major carrier of S1P, an endothelial-barrier stabilizing agent in blood. In humans, plasma S1P closely correlates with Hct suggesting that RBC-associated S1P is in close equilibrium with plasma S1P. We did not find a similar relationship in mice, which is in keeping with other observations [12]. In fact, observations of higher RBC-associated S1P levels after blood loss suggest that mice have a more robust homeostatic mechanism to replenish circulating S1P when Hct declines. Hla and colleagues have suggested that endothelial cells may be a major contributor to blood S1P levels in mice [12]. Their observations, together with our own, support a model in which RBC-S1P is in equilibrium with endothelial (or other) sources of S1P, and in setting of blood loss, RBC-associated S1P is rapidly replenished from these sources. 

We recently demonstrated that S1P levels decline markedly in human RBCs during storage as a result of enzymatic degradation. In this paper, we observed a similar decline in murine RBC-S1P levels during storage and a lowering in blood S1P after transfusion of aged RBCs. A lowering of S1P carrying capacity of aged RBC might explain our previous observations that RBC transfusion in humans had variable effects on restoration of plasma S1P and the trend towards an inverse correlation between RBC storage duration and posttransfusion increase in plasma S1P [8]. 

Sphingosine-1-Phosphat has a potent and unique barrier-protective property in cultured endothelial cells [13, 14] and intact microvessels [15]. In these systems, administration of S1P reverses the vascular permeability and inflammatory lung injury induced by LPS [16, 17] and ischemia-reperfusion injury [18], although toxicity occurs at high concentrations of S1P [19]. The loss of RBC-associated S1P during storage may explain in part the observation that aged RBCs in anemic mice that were given LPS resulted in increased lung permeability in comparison to mice transfused with freshly isolated RBCs. To explore this possibility, we tested the ability of S1P-loaded RBCs to prevent pulmonary vascular leak following transfusion. We found that mouse RBCs accumulated exogenous S1P and transfusion of S1P-loaded fresh or aged RBC, increased plasma S1P levels, and protected against the barrier-destabilizing effects of transfusion observed in the setting of anemia and LPS challenge. The precise mechanisms involved are presently unclear but could involve improved exposure of the microcirculation to activating levels of S1P to maintain lung endothelial-barrier integrity. 

## 5. Conclusion

In conclusion, loss of S1P associated with storage of RBCs may contribute to blood transfusion-related pulmonary vascular leak in the setting of acute anemia and systemic inflammation. Transfusion of exogenous S1P-loaded RBCs can partly attenuate acute pulmonary vascular leak. Our findings could have clinically important ramifications for novel therapeutic strategies to predict and prevent for transfusion-related injury.

## Figures and Tables

**Table 1:** Platelet number and function.

Condition	Platelet count (×10^3^/*μ*L)	ADP aggregation (AUC)	PAR4 aggregation (AUC)
Baseline	670 ± 115	32 ± 17	48 ± 2
Anemia	566 ± 184	33 ± 25	48 ± 12
Anemia + LPS	399 ± 164	30 ± 36	14 ± 13^∗^
LPS	350 ± 137^∗^	21 ± 12^∗^	17 ± 14^∗^
Anemia + RBC	721 ± 187	34 ± 17	53 ± 4
Anemia + aged RBC	374 ± 95	37 ± 24	38 ± 14

^∗^ *P* < 0.05 versus baseline by one-way ANOVA with Bonferroni correction.

## Abbreviations

BSA: Bovine serum albumin
CBC: Complete blood count
CPD: Citrate phosphate dextrose solution
CXCL1/KC: Keratinocyte-derived chemokine
DHS1P: Dihdro-sphingosine-1-phosphate
EBD: Evans blue dye
Hct: Hematocrit
IL-6: Interleukin 6
LPS: Lipopolysaccharide
PBS: Phosphate buffered saline
OD: Optical density
RBC: Red blood cell
S1P: Sphingosine-1-phosphate.

## References

[1] Repine JE (1992). Scientific perspectives on adult respiratory distress syndrome. *The Lancet*.

[2] Shenkar R, Abraham E (1997). Hemorrhage induces rapid in vivo activation of CREB and NF-*κ*B in murine intraparenchymal lung mononuclear cells. *American Journal of Respiratory Cell and Molecular Biology*.

[3] Parsey MV, Tuder RM, Abraham E (1998). Neutrophils are major contributors to intraparenchymal lung IL-1*β* expression after hemorrhage and endotoxemia. *Journal of Immunology*.

[4] Chapman CE, Stainsby D, Jones H (2009). Ten years of hemovigilance reports of transfusion-related acute lung injury in the United Kingdom and the impact of preferential use of male donor plasma. *Transfusion*.

[5] Vlaar APJ, Straat M, Juffermans NP (2011). The relation between aged blood products and onset of transfusion-related acute lung injury: a review of pre-clinical data. *Clinical Laboratory*.

[6] Tung J-P, Fraser JF, Nataatmadja M (2012). Age of blood and recipient factors determine the severity of transfusion-related acute lung injury (TRALI). *Critical Care*.

[7] Bode C, Sensken SC, Peest U (2010). Erythrocytes serve as a reservoir for cellular and extracellular sphingosine 1-phosphate. *Journal of Cellular Biochemistry*.

[8] Selim S, Sunkara M, Salous AK (2011). Plasma levels of sphingosine 1-phosphate are strongly correlated with haematocrit, but variably restored by red blood cell transfusions. *Clinical Science*.

[9] Sadahira Y, Yasuda T, Yoshino T (2000). Impaired splenic erythropoiesis in phlebotomized mice injected with CL2MDP-liposome: an experimental model for studying the role of stromal macrophages in erythropoiesis. *Journal of Leukocyte Biology*.

[10] Beutler E, West C, Blume KG (1976). The removal of leukocytes and platelets from whole blood. *Journal of Laboratory and Clinical Medicine*.

[11] Hänel P, Andréani P, Gräler MH (2007). Erythrocytes store and release sphingosine 1-phosphate in blood. *FASEB Journal*.

[12] Venkataraman K, Lee YM, Michaud J (2008). Vascular endothelium as a contributor of plasma sphingosine 1-phosphate. *Circulation Research*.

[13] Dudek SM, Jacobson JR, Chiang ET (2004). Pulmonary endothelial cell barrier enhancement by sphingosine 1-phosphate. Roles for cortactin and myosin light chain kinase. *The Journal of Biological Chemistry*.

[14] Garcia JGN, Liu F, Verin AD (2001). Sphingosine 1-phosphate promotes endothelial cell barrier integrity by Edg-dependent cytoskeletal rearrangement. *Journal of Clinical Investigation*.

[15] McVerry BJ, Peng X, Hassoun PM, Sammani S, Simon BA, Garcia JGN (2004). Sphingosine 1-phosphate reduces vascular leak in murine and canine models of acute lung injury. *American Journal of Respiratory and Critical Care Medicine*.

[16] Peng X, Hassoun PM, Sammani S (2004). Protective effects of sphingosine 1-phosphate in murine endotoxin-induced inflammatory lung injury. *American Journal of Respiratory and Critical Care Medicine*.

[17] Szczepaniak WS, Zhang Y, Hagerty S (2008). Sphingosine 1-phosphate rescues canine LPS-induced acute lung injury and alters systemic inflammatory cytokine production in vivo. *Translational Research*.

[18] Okazaki M, Kreisel F, Richardson SB (2007). Sphingosine 1-phosphate inhibits ischemia reperfusion injury following experimental lung transplantation. *American Journal of Transplantation*.

[19] Sammani S, Moreno-Vinasco L, Mirzapoiazova T (2010). Differential effects of sphingosine 1-phosphate receptors on airway and vascular barrier function in the murine lung. *American Journal of Respiratory Cell and Molecular Biology*.

